# Electrically tunable metasurface by using InAs in a metal–insulator–metal configuration

**DOI:** 10.1515/nanoph-2021-0618

**Published:** 2022-02-02

**Authors:** Junghyun Park, Seong Jun Kim, Volker J. Sorger, Soo Jin Kim

**Affiliations:** Samsung Advanced Institute of Technology, Samsungro 130, Youngtong-gu, Suwon 16678, Republic of Korea; School of Electrical Engineering, Korea University, Engineering Building Room #216,145, Anam-ro, Seongbuk-gu, Seoul 02841, Republic of Korea; Department of Electrical and Computer Engineering, George Washington University, Science & Engineering Hall, 800 21st Street, NW, ​Washington DC 20052, USA

**Keywords:** InAs, metasurface, optical modulator, surface plasmonics, wavefront shaping

## Abstract

The ability of modulating optical properties at a lateral subwavelength scale is of crucial importance due to its potential applications for wide-angle holographic displays, optical communications, and interferometric sensors. Here, we present an electrically tunable metasurface whose optical properties can be element-wise controlled at the lateral subwavelength scale in the mid-infrared wavelength regime. Our proposed device facilitates an *n*-doped InAs layer as a dynamic-tunable layer, and the charge carrier concentration inside the InAs layer is tailored by external gate biases. This InAs active layer is sandwiched between top aluminum strip antennas and a bottom gold substrate, forming the metal–insulator–metal configuration. The change of the charge carrier concentration gives rise to modulation of the amplitude and phase of reflected light in a mid-infrared regime. Numerical investigations show the reflectivity contrast of 44%P with biases of −2.5–0 V and the phase change of 236° with biases of −15 V to +15 V at the wavelength of ∼5 μm. Versatile wavefront shaping such as beam focusing with Fresnel Zone plate and beam steering with saw-tooth phase grating is also provided.

## 1 Introduction

Optical modulators are devices that convert signals contained in external forms such as electrical voltages into optical information. Such information can be represented by temporarily or spatially varying optical amplitude or phase of a guided optical mode or a plane wave in free space. In particular, the optical modulators in the free space, which are also known as a spatial light modulator (SLM) [1], play important roles in display [2] and optical sensor [3]. Various tunable mechanisms have been studied and developed for such SLMs, such as liquid crystal [4] and micro-electro-mechanical systems (MEMS) [5].

Meanwhile, there has been considerable research on implementation of novel SLMs with a fine pixel pitch and a fast operation speed [6], [7], [8], [9]. The fine pixel pitch enables a wide field of view, and the fast operation in order of MHz allows for novel optical sensors such as scanning beam microscopy [10] and light detection and ranging [11]. Transparent conducting oxides such as an indium-tin-oxide (ITO) have been exploited for electrically tunable metasurface that shows ∼8%P reflectivity modulation [12] and around 180°–210° phase change in the near and mid-infrared wavelength regimes [13], [14], [15], [16]. By tailoring chemical potential of the Fermi energy in a single layer graphene placed in plasmonic structures, substantial reflection phase changes have been demonstrated [17], [18], [19], [20]. To enhance the extent of tuning, various phase-change materials with gigantic index-changing effect such as GeSbTe and VO_2_ have been investigated [21], [22], [23], [24]. Recently, it was also shown that highly doped III-V semiconductors such as InAs and GaN/AlGaN can be employed for a dynamic control of a thermal emission [25, 26].

The fundamental bottleneck of utilizing those electrically tunable metasurfaces for practical applications is a low efficiency. For example, metasurfaces that facilitate ITO in plasmonic resonators have reported the efficiency around 1–3% [13], [14], [15]. This small efficiency comes from a limited modulation depth due to a non-negligible dissipation in the ITO and a resultant low *Q*-factor of the resonators. One of the authors have also examined the use of a low-doped InAs layer, as a replacement of ITO, sandwiched between top Al gratings and a high-doped InAs substrate. The strengths of this approach are that the low-doped InAs layer has a dissipation much smaller than that of ITO, and that an effective mass of InAs is considerably small and thus the plasmon frequency can be changed significantly for the given range of charge concentration change. However, this configuration adopted the highly doped InAs layer as a bottom mirror because of the epitaxial growth of the low-doped active InAs layer. Since a magnitude of the negative-valued real part of highly doped InAs layer is small at the wavelength of interest, gap plasmons inside the resonators exhibit deep penetration into the highly doped InAs mirror. Consequently, the modulation depth of the configuration is small.

Here, we present an electrically tunable metasurface that exploit the low-doped InAs layer as an active layer sandwiched between the top and bottom metallic structures. The core novelty is that we assume to replace the highly doped InAs mirror with a metallic (Au) substrate by using the bonding process. The large magnitude of the negative-valued real part of Au allows negligible penetration depth, and thus the mode confinement of the gap plasmons is remarkably increased. In addition, by judiciously designing the grating width and the spacing, i.e., the edge-to-edge distance between neighboring gratings, it is possible to align a resonance wavelength of the gap plasmon with a material resonance wavelength of *n*-doped InAs; the epsilon-near-zero (ENZ) wavelength, and to set a desired coupling dynamics. By using electrical simulations for the charge distribution and full-field optical simulations, we show the reflectivity change of 44%P in an amplitude modulation scheme and the reflection phase change of 236° in a phase modulation scheme.

Each section of this report is organized as follows. We describe the proposed structure and the working principle in Section 2. Then, its operation as the amplitude-modulator is demonstrated, where the dynamic focusing is achieved by using Fresnel Zone plate. In Section 4, we present the phase-modulation scheme and the beam steering via saw-tooth phase profile, then conclude in Section 5.

## 2 Structure and working principle

**Figure 1:**
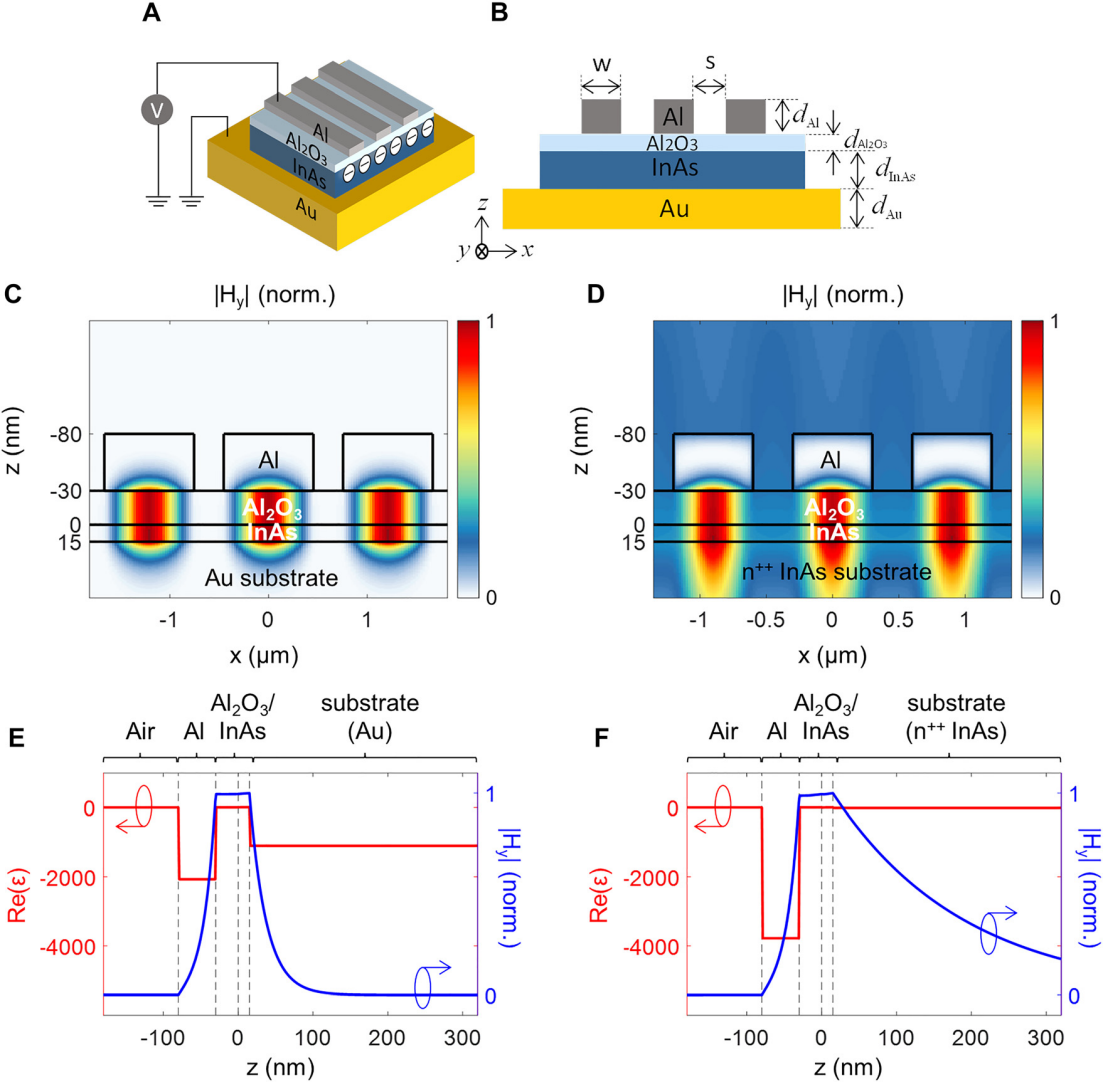
Configuration of the proposed metasurface with a strong light field confinement. (A) Bird’s eye-view of the metasurface including three Al antenna on top of an Al_2_O_3_ layer as a dielectric insulator and an *n*-doped InAs layer as an active layer, where the charge carrier concentration at the interface between the Al_2_O_3_ and InAs layers can be controlled to manipulate the amplitude and phase of reflected light. (B) Cross-sectional view with the detailed design parameters. *d*_Al_: 50 nm, *d*_InAs_: 15 nm in common. *d*_Au_ is assumed to be semi-infinite. *w*, *s*, *d*_Al2O3_ are 660 nm, 50 nm, and 5 nm, respectively, for the amplitude modulation, and 910 nm, 30 nm, and 30 nm, respectively, for the phase modulation. (C) Magnitude of the magnetic field distribution at a resonance of the gap plasmon mode formed between the top Al strip antenna and the bottom Au substrate. *w*, *s*, *d*_Al2O3_ are 910 nm, 300 nm, and 30 nm. The wavelength is 4.7 μm, and the incident beam has the normal angle and the electrical field perpendicular to Al strip antennas. We observe a gigantic field confinement in the cavity made of InAs layer and a negligible penetration into the Au substrate. (D) Magnitude of magnetic field distribution for the case with the high-doped (*n* ^++^) InAs substrate as in Ref. [25] (*N*_d_ = 10^20^/cm^3^) (see Table 1 for details). *w*, *s*, *d*_Al2O3_ are 600 nm, 300 nm, and 30 nm. The wavelength is 6.6 μm. It is clearly seen that the gap plasmon exhibits longer penetration into the substrate compared to (C). (E) Spatial profile of the real part of permittivity (red line, left *y*-axis) and the normalized magnitude of the *y*-component of the magnetic field (blue line, right *y*-axis) in the presence of the Au substrate in (C), showing small penetration depth to the substrate and strong light field confinement at the core. (F) Spatial profile for the high-doped substrate as in (D), showing substantial penetration depth to the substrate and thus lower light field confinement at the core.

We illustrate a schematic diagram of the proposed configuration in Figure 1A. An *n*-doped InAs layer is bonded on top of Au. We chose Au due to its small skin depth and low optical loss in the mid-infrared regime. We assume a doping concentration of the InAs layer as *N*_D_ = 10^19^/cm^3^. Figure 1B shows a cross-sectional view of the proposed configuration. The thickness of the *n*-doped InAs layer is set *d*_InAs_ = 15 nm. The Au substrate with semi-infinite thickness plays roles both of an optical mirror and an electrical ground. The InAs layer is used as an active layer, the optical constant of which can be tuned by a top gate. To implement this gate structure, a dielectric insulator of Al_2_O_3_ is placed on top of the InAs layer. The advantage of using alumina is high dielectric strength of 7.3 MV/cm and large DC permittivity of ∼7.74 [25]. These beneficial properties contribute to a substantial change in the charge carrier concentration at an interface between the InAs layer and the Al_2_O_3_ layer, resulting in an improved modulation depth. The thickness of the alumina layer is designed as *d*_Al2O3_ = 5 nm and 30 nm for the amplitude modulation and the phase modulation, respectively. The maximum magnitude of the applied electric field is set to 5 MV/cm, which corresponds to 2.5 V for 5 nm and 15 V for 30 nm, respectively. As a final step, Al gratings with the thickness *d*_Al_ = 50 nm are made on top of the dielectric insulator. The width and the period of the Al gratings vary depending on a purpose or desired functionality, which will be discussed below.

Normally incident light with an electric field perpendicular to the grating is coupled to a gap plasmon mode in the Al/Al_2_O_3_/InAs/Au configuration. The gap plasmon mode propagates through a core formed by Al_2_O_3_/InAs and is reflected back at an end facet of the Al strip with a reflection phase pick-up, as depicted in a cross-sectional view in Figure 1B. If the round-trip phase is an integer multiple of 360°, a resonance occurs, leading to a dip in a reflection spectrum. Figure 1C shows the *y*-component (out of the plane) of the magnetic field distribution at a resonance. It is clearly seen that a strong magnetic dipole is induced at the center of the strip cavity. The incident energy is resonantly coupled to the magnetic dipole, which gives rise to a dip in a reflection spectrum.

A distinguished approach of this study is that we employ the Au as a substrate, whereas previous related research exploited a highly doped (*n* ^++^) InAs layer (*N*_d_ = 10^20^/cm^3^) as a substrate. The substitution of the substrate from the *n* ^++^ InAs to Au is advantageous in terms of a modulation depth. This is because a larger negative-valued magnitude of the real part of the permittivity of Au contributes to a gap plasmon mode with a more confined electromagnetic field at the core with the active material. The magnetic field distribution assuming the highly doped InAs substrate is shown in Figure 1D. Comparing Figure 1C and D, we clearly observe that the InAs substrate allows longer penetration into the substrate. This gives rise to substantial loss in the modulation.

In Figure 1E, we plot the real part of the relative electric permittivity (Re(*ε*), left *y*-axis) and the *y*-component of the magnetic field (right *y*-axis) of the gap plasmon modes in the structure for the Au substrate case (Figure 1C). The Re(*ε*) of the Al grating is around −2068, and that of the Au substrate is around −1099 at the wavelength of 4.7 μm. The mode index of the gap plasmon is around 2.39, and the penetration depth into the Au substrate is 22.3 nm. As a result, the light field is mostly confined at the core part, and such a large overlap between the electromagnetic field and the active (index-changing) layer facilitates efficient modulation, which is extremely beneficial to amplitude and phase control.

In contrast, the case of the highly doped InAs substrate (Figure 1F) clearly shows longer evanescent tail into the InAs substrate due to its small magnitude of the metallic cladding (negative-valued Re(*ε*) of the highly doped InAs substrate). The mode index here is around 5.32, and the penetration depth into the highly doped InAs substrate is 173 nm at the wavelength of 6.6 μm. Consequently, the light field confinement at the core is weak, and thus modulation depth is smaller than the case of the Au substrate.

To investigate effect of electrical biases on optical responses, we examine the electron carrier concentration as a function of the gate and the corresponding permittivity. The permittivity ϵ is described by the Drude’s model as follow [27]:

(1) $$\epsilon =\epsilon_{inf}-\frac{\omega_{P}^{2}}{\omega \left(\omega +1i\Gamma \right)}$$

where $\epsilon_{inf}$ denotes the infinite frequency permittivity, ω is the angular frequency, Γ is the collision frequency, and *i* is the square root of −1. $\omega_{P}$ is the plasmon frequency, and is given by

(2) $$\omega_{P}=\sqrt{\frac{Ne^{2}}{\epsilon_{0}m_{e}^{*}}}$$

where *N* is the charge carrier concentration, e is the charge of an electron, $\epsilon_{0}$ is the free space permittivity, and $m_{e}^{*}$ is the effective mass of an electron. We invoke each value for electrical bias based on our previous study in which the parameters are empirically obtained by fitting numerical model with the measured data: the effective electron mass of $m_{e}^{*}$ = 0.0394 $m_{e}$ , where is $m_{e}$ the electron mass [25] (please see Table 1).

**Table 1:** Parameters used for the Drude’s model with the charge concentration (*N*) from the electrical simulation and the material properties ( $\epsilon_{inf}$ , $m_{e}^{*}$ / $m_{e}$ , and Γ ) from the previous empirical study in [25].

Electric field	$\epsilon_{inf}$	N	$m_{e}^{*}$ / $m_{e}$	$\omega_{P}$	Γ
InAs (5 MV/cm)	12	1.0000 × 10^20^/cm^3^	0.0394	2.84 × 10^15^ rad/s	2.0 × 10^13^ rad/s
InAs (0 V)	12	1.0000 × 10^19^/cm^3^	0.0394	8.99 × 10^14^ rad/s	2.0 × 10^13^ rad/s
InAs (−5 MV/cm)	12	0	0.0394	0	2.0 × 10^13^ rad/s
Au	9	2.0943 × 10^22^/cm^3^	0.3500	1.38 × 10^16^ rad/s	1.094 × 10^14^ rad/s

**Figure 2:**
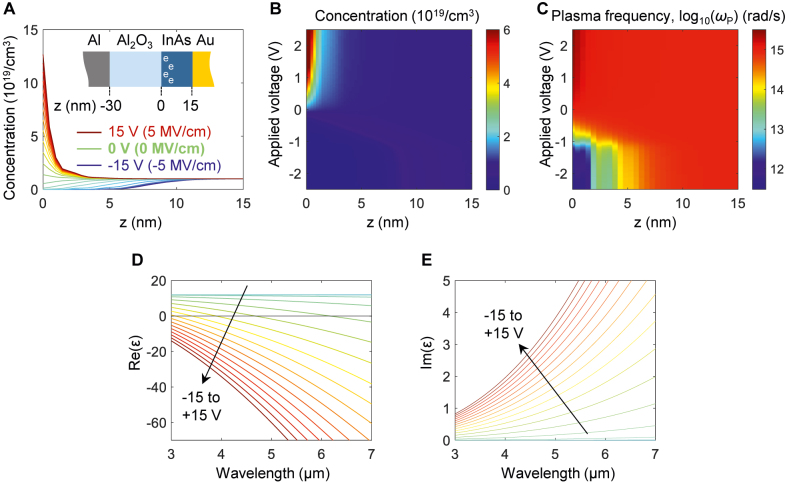
Optical property of the InAs described by the Drude’s model. (A) Charge carrier concentration distribution for various voltage values. The inset shows the schematic distribution of the Al/Al_2_O_3_/InAs/Au configuration. (B) Charge carrier concentration map for the position and the applied bias. (C) Plasma frequency map in a log scale obtained from (B) and Eq. (2). (D) Real part of the relative electric permittivity at the interface between Al_2_O_3_ and InAs layers (*z* = 0) for the wavelength range from 3 μm to 7 μm for the different bias cases of the depletion (blue, −5 MV/cm), the no-bias (green, 0 MV/cm), and the accumulation (red, 5 MV/cm). (E) Imaginary part of the relative electric permittivity, showing the negligible optical loss in the depletion case (blue) and significant increase of the optical loss in the accumulation case (red).

By using the electrical simulation (Lumerical CHARGE™), we calculate the exact charge carrier concentration distribution inside the 15 nm-thick InAs active layer for the applied voltage. We show the electrical simulation results in Figure 2A. Here, *d*_Al2O3_ is assumed to be 30 nm and thus the voltage ranges from −15 V (−5 MV/cm) to +15 V (+5 MV/cm). The case with *d*_Al2O3_ of 5 nm was also calculated for the voltages ranging from −2.5 V (−5 MV/cm) to +2.5 V (+5 MV/cm), which are the same as in *d*_Al2O3_ of 30 nm.

In Figure 2A, the case with 0 V (no-bias) shows quasi-flat concentration of *N* = 10^19^/cm^3^ across the InAs layer (green line). As we apply the negative bias, the depletion region is formed. At the maximum negative bias with the voltage of −15 V (−5 MV/cm), we observe wide depletion region with the almost 6 nm (blue line). On the other hand, the positive bias leads to the strong charge concentration at the interface between the Al_2_O_3_ and InAs layers. The maximum positive bias with the voltage of +15 V (+5 MV/cm) gives rise to the charge concentration of more than 10^20^/cm^3^ at *z* = 0.

By combining the charge concentration distribution across the *z*-coordinate for the given voltage (Figure 2B) and Eq. (2), we can obtain the plasma frequency. Figure 2C shows the distribution of $\omega_{P}$ in the log scale. We note that the change of the concentration results in significant change in the plasma frequency. This can be ascribed to the small effective mass of free electrons inside InAs, which is around 0.0394 × *m*_e_. This is an order of magnitude small than that of the indium tin oxide (0.45 × *m*_e_) [12]. Therefore, the use of InAs as an active layer is advantageous with the larger changes in the plasma frequency for the given carrier concentration change.

As a final step, we invoke the Drude’s model (Eq. (1)) to generate the permittivity change. Since the electrical permittivity is a function of not only the voltage and *z*-coordinate, but also a function of the wavelength, we pick the *z*-coordinate at the interface between the Al_2_O_3_ and InAs layers (*z* = 0) for the sake of visualization. Figure 2D shows the real part of the relative electrical permittivity in the wavelength regime of interest. The blue and red curves denote the permittivity for the maximum negative (−15 V) and positive (+15 V) voltages, respectively. The cyan, green, and yellow curves correspond to those at the intermediate values with the voltage in the increasing direction denoted by the black arrow. Note that the blue and cyan curves are overlapped; it is hard to distinguish one from another, which can be ascribed to the limited range in the wavelength (3–7 μm). The green line corresponds to the case of no-bias. It exhibits monotonically decreasing behavior as the wavelength increases. We note that it crosses a line of zero at the wavelength of 5.0 μm. This is referred to as the epsilon-near-zero (ENZ) wavelength. Since the electric flux density normal to an interface is continuous across the interface, the normal electric field, here z-component, is significantly enhanced at the ENZ wavelength. This phenomenon contributes to significantly overlap between the electric field and the active material, which is especially advantageous for efficient modulation [12].

Now let us examine the effect of the bias. We consider both cases of the electrical biases with a positive sign for formation of an accumulation layer and a negative sign for formation of a depletion layer. The former is depicted by the red curve in Figure 2D. The increased charge carrier concentration leads to the rise of the plasma frequency as in Eq. (2). According to the Drude’s model described in Eq. (1), this decreases the real part of the electric permittivity. As a result, the resonance wavelength exhibits a blue-shift. In contrary, the formation of the depletion layer results in the decrease of the plasma frequency and a corresponding red-shift. By combining these spectra shifts, it is possible to achieve substantial modulation in the amplitude, or the intensity. Not only the electrical bias to the InAs layer allows the modulation of the real part of the electrical permittivity, but it also enables the control of the imaginary part. This is because of non-negligible collision frequency in Eq. (1) and Table 1. Figure 2E plots the imaginary part of the electrical permittivity. The notation and the condition of each curve is the same as in Figure 2D. It is observed that the imaginary part of the electrical permittivity is increased in the accumulation case (the red curve) and decreased in the depletion case (the blue curve), compared to the no-bias case (the green curve). This ability enables dynamically tuning of the dissipation of the gap plasmon mode, and thus allows for large phase change as will be shown below.

## 3 Amplitude modulation

We demonstrate the ability of the proposed tunable metasurface for amplitude modulation. The optimum designs to maximize the extent of contrast in amplitude are provided. The key design parameters are the width of each strip and spacing, i.e., edge-to-edge distance between neighboring strips, as they determine the resonance wavelength and the coupling dynamics.

**Figure 3:**
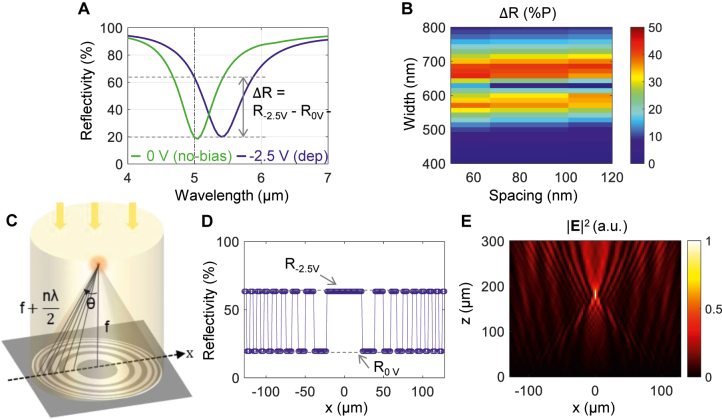
Amplitude modulation and beam focusing of mid-infrared light. (A) Reflectivity spectrum for different voltages of 0 V (the green line) for no-bias, and −2.5 V (the blue line) for depletion. The contrast of the reflectivity under applied biases denoted by Δ*R* is the maximum at the wavelength of 5.0 μm denoted by the vertical dash-dotted line. (B) Map for the reflectivity contrast for different values of widths and spacings. (C) Schematic of the Fresnel Zone plate consisting of multiple co-centered rings with alternating intensity. (D) Reflectivity distribution assigned to each Al antenna to implement the Fresnel Zone plate in (C) as an example of beam focusing with the focal length of 200 μm according to (2). (E) Electric field intensity distribution calculated by using the one-dimensional variation of the reflectivity given in (D), indicating that the focus is formed at the designed focal length of 200 μm.

Figure 3A shows the reflectivity spectrum for the no-bias (0 V) depicted by a green curve and the depletion (−2.5 V) shown with a red curve. The results from the electrical simulation described in Figure 2 are plugged into the full-field optical simulation with the stair-case approximation. We divided the 15 nm-thick InAs layer into 30 sub-layers along the *z*-coordinate, which leads to the unit thickness of 0.5 nm. For each layer, we calculated the carrier concentration, the corresponding plasma frequency, and the electrical permittivity for each wavelength.

The grating width is 660 nm and the spacing is 50 nm, indicating that the period is 710 nm *d*_Al2O3_ is 5 nm. The reflectivity contrast Δ*R* defined as the difference between the reflectivity at the no-bias (*R*_0V_) and the depletion (*R*_−2.5V_) reaches its maximum of Δ*R* = 44%P, where %P is the percentage point describing the difference of two percentage values, at the wavelength of 5.0 μm (*R*_0V_ = 19.5% and *R*_−2.5V_ = 63.4%). Since the transmission is prohibited due the presence of opaque material, Au here, the absorption is directly complementary of the reflection, i.e., *A*_0V_ = 80.5% and *A*_−2.5V_ = 36.6%, where A_0V_ denoted the absorption at the no-bias, and *A*_−2.5V_ indicates that at the depletion. The reflectivity spectrums for other voltages and the effect of geometric parameters are provided in Supplementary Note 1.

To understand the functional behavior of the large reflectivity contrast, we calculate Δ*R* for various grating width and spacing, and show the result in Figure 3B. It is noticeable that the maximum reflectivity occurs around the grating width of 660 nm. We also note that the effect of the spacing on the contrast is not significant. We picked a spacing value as 50 nm.

With spatially varying intensity contrast, we can achieve focusing with variable focal length. A device that utilizes this effect is called the Fresnel Zone plate (Figure 3C). A Fresnel Zone plate is composed of multiple co-centered rings with alternating transparency; opaque and transparent rings in an ideal case. The pitch of these rings is gradually decreased as the ring radius increases [28].

(3) $$r_{n}=\sqrt{m\lambda f+\frac{1}{4}m^{2}\lambda^{2}},$$

where *m* is an integer and *f* is the focal length. As a result, beams incident on the outside region are deflected with larger angles. At the focal position, the deflected beams merge, or constructively interfere on the optical axis, and this point is called the focus.

Figure 3D shows the reflectivity that are assigned to each antenna with the grating width 660 nm and the spacing 50 nm. The focal length is set to be 200 μm. We use two discrete of the maximum ( $R_{-2.5V}$ ) and the minimum ( $R_{0V}$ ) reflectivity that are alternatively modulated along the *x*-axis according to the binary Fresnel zone plate design as follow: [28].

(4) $$R\left(x\right)=\left\{ \begin{array}{l} R_{acc},if\frac{1+sign\left(\cos\left(\frac{2\pi x^{2}}{\lambda_{0}f}\right)\right)}{2}=1\\ R_{dep},if\frac{1+sign\left(\cos\left(\frac{2\pi x^{2}}{\lambda_{0}f}\right)\right)}{2}=0 \end{array}\right.$$

By assuming normal incidence with the wavelength of 5.0 μm and the transverse magnetic (TM) polarization, we calculate the intensity distribution of the reflected beam and show the result in Figure 3E. It is noticeable that the focus is formed at (*x*, *z*) = (0 μm, 200 μm), which agrees well with the theoretical prediction. By controlling the focal position *f* in (2), it is possible to arbitrarily control the focus. The reflectivity and the field distributions for other focal positions of 100 μm and 400 μm are provided in Supplementary Note 2 (Figure S3).

## 4 Phase modulation

The proposed metasurface can also be applied to phase modulation, and the spatially varying phase distribution enables on-demand beam steering. To verify the ability to tune the reflection phase pickup, we designed the grating to maximize the phase range that can be covered under the electrical bias. For this goal we reduce the spacing of the grating. The incident beam experiences narrow gap, and the lateral wavevector of the transmitted beam is enhanced due to the increased diffraction. Consequently, the coupling ratio of light into the strip cavity increases, which gives rise to balance between the coupling rate and the dissipation rate. We chose the grating width of 910 nm, the spacing of 30 nm, which turned out to enable the maximum phase change at 5.16 μm. The thickness of Al_2_O_3_ oxide layer is chosen to be 30 nm to ensure the operation near the critical coupling. In this case the capacitance of the device with the size of 100 × 100 μm^2^ as 21.8 pF. Assuming the resistance of the device as 100 Ω [25], the operation speed of our configuration can be estimated as 3 dB cut-off frequency of 72.9 MHz, which may facilitate ultrafast dynamic beam shaping for various time-limited applications.

**Figure 4:**
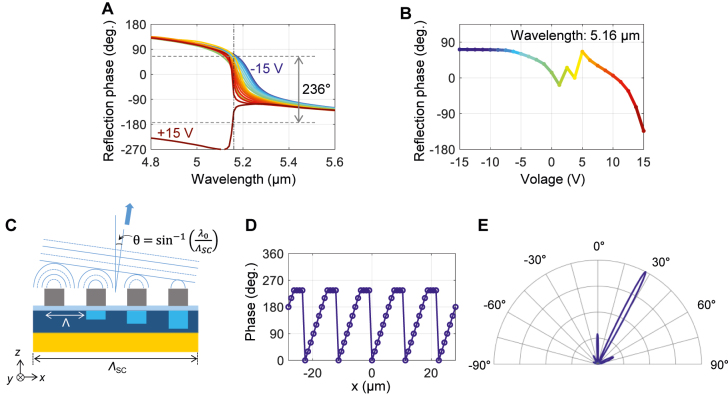
Phase modulation and beam steering of mid-infrared light. (A) Reflection phase spectrum for different voltages of −15 V (the blue line) to +15 V (the red line), showing the transition from the over-coupling (the blue line) to the under-coupling (the red line) regimes. (B) Relative change of the reflection phase at the wavelength of 5.16 μm denoted by the vertical dash-dotted line in (A), showing the continuous phase sweep up to 236°. (C) Schematic cross-sectional view of the phase-tunable metasurface, where we assume that each Al antenna is fed by a proper voltage to produce a saw-tooth phase gradient. The steering angle *θ* can be controlled by changing $\Lambda_{\text{SC}}$ . (D) An example of phase distribution with super cells composed of 12 antennas. (E) Far-field intensity as a function of the angle, showing well-defined main lobe at the designed angle of 27.2°.

Figure 4A shows the reflection phase spectrum for the electrical bias from −15 V (blue, depletion) to +15 V (red, accumulation), with an incremental voltage step of 1.25 V (25 cases in total). At the bias with +12.5 V, the coupling dynamics is near the critical-coupling [29]. First, as we further induce the accumulation layer with larger voltages, the charge concentration in InAs is also increased. As a result, the coupling dynamics moves to the under-coupling regime, which can be observed from the swinging phase spectrum as in Figure 4A. Second, as we induce the depletion with the negative bias, the charge concentration is decreased, and the material loss becomes negligible in InAs. Consequently, the coupling dynamics moves to the over-coupling regime, as shown in Figure 4A. Due to this transition of the coupling dynamics, it is possible to achieve large phase change even with the limited modulation depth. The quantitative analysis of the dissipation and coupling rate of each bias state is summarized in Table 2.

**Table 2:** Dissipation and coupling rates of each bias state by using the coupled mode theory in [29].

Bias state	Dissipation rate	Coupling rate	Dynamics
−15 V (depletion)	1.2494 × 10^13^ rad/s	1.5129 × 10^13^ rad/s	Over-coupling
+12.5 V (accumulation)	1.3620 × 10^13^ rad/s	1.4410 × 10^13^ rad/s	Critical-coupling
+15 V (accumulation)	1.4159 × 10^13^ rad/s	1.3871 × 10^13^ rad/s	Under-coupling

To visualize relationship between the applied bias and the phase change, we plot the reflection phase in Figure 4B. At the wavelength of 5.16 μm, the phase change covers 236°. The range with the phase change over 180° is around 20 nm (Figure S4 in Supplementary Note 2). We note that the phase change can even further extended by employing high-k insulator such as hafnium aluminum oxide laminate due to improved electrostatics [15].

Based on the established relationship or look-up table in Figure 4B, we can apply required voltage distribution in the antenna array to have phase gradient for beam steering. Figure 4C shows the schematic diagram of the phase-tunable metasurface with saw-tooth phase gradient. Each spherical wavelet generated by individual antenna form an equi-phase surface, normal to which the reflected beam is deflected. By adjusting distribution of the phase gradient to control the slope of this wavefront, it is possible to achieve beam steering with arbitrary angle.

Figure 4D shows an example of the phase distribution from each antenna. We aim to generate saw-tooth phase gradient to steer beam to the angle of 27.2°. The required equi-phase surface can be generated by using 12 antennas as a super cell. The phase of each super cell varies periodically across the group due to the 360° unwrapping, and $\Lambda_{\text{SC}}$ is defined as the period of the super cell. The deflection angle is determined by the relationship between the wavelength $\lambda_{0}$ and the super cell period $\Lambda_{\text{SC}}$ as follow [28]:

(5) $$\theta =\sin^{-1}\frac{\lambda_{0}}{\Lambda_{\text{SC}}}$$

We take the limitation in the maximally achievable phase change into account and assign the phase value of 236° to antennas that requires phase above 236°. By changing the number of antennas in a super cell, it is possible to change deflection angle (Figure S4 in Supplementary Note 2). We assumed the device length is 56.4 μm, composed of 60 Al antennas with the period of 940 nm. At the wavelength of 5.16 μm, the beam divergence is calculated as ∼3°. In Figure 4E, the far-field intensity versus the deflection angle is plotted, in which good agreement between theory and simulation is observed.

## 5 Conclusions

We presented an electrically tunable metasurface that utilizes an *n*-doped InAs layer as an active material sandwiched between plasmonic Al strip antennas and the Au substrate. Applying electrical biases induces accumulation and depletion of charge carrier concentration inside the InAs layer, which in turn leads to control of the dielectric constant. Numerical investigations have demonstrated that we can achieve the amplitude modulation with gigantic intensity contrast up to 44%P in the reflection as well as the phase modulation with significant phase range up to 236° by using judiciously designed Al strip antennas with various widths and spacings. It turned out that these advantageous performances come from the strong confinement of electromagnetic field inside the index-changing material due to the presence of metallic substrate and antennas as well as the low effective electron mass of the InAs layer. We believe that the proposed configuration may pave the way for the development of high-efficiency free-space spatial light modulators that may find their wide applications such as holographic display, beam steering, and optical communications.

## Supplementary Material

Supplementary Material
