# Supplementary material for: Electrically tunable metasurface by using InAs in a metal–insulator–metal configuration
Source: Nanophotonics. 2022 Feb 2;11(6):1117–26. doi: 10.1515/nanoph-2021-0618 (PMC11501525; doi:10.1515/nanoph-2021-0618)
Supplement: Supplementary file 1 — Supplementary Material [file j_nanoph-2021-0618_suppl.docx]

## Supplementary Information

Electrically tunable metasurface by using InAs in a metal-insulator-metal configuration

Junghyun Park^1,§^, Seong Jun Kim^2,§^, Volker J. Sorger^3^, and Soo Jin Kim^2,*^

^1^Samsung Advanced Institute of Technology, Samsungro 130, Youngtong-gu, Suwon, 16678, Rep. of Korea

^2^School of Electrical Engineering, Korea University, Engineering Building Room #216,145, Anam-ro, Seongbuk-gu, Seoul, 02841, Rep. of Korea

^3^Department of Electrical and Computer Engineering, George Washington University, Science & Engineering Hall, 800 21^st^ Street, NW, ​Washington DC, 20052, USA

Corresponding author: *kimsjku@korea.ac.kr

^§^These authors contributed equally to this work.

**Supplementary Note 1.** Parametric analysis and optimization of InAs metasurface for reflectivity modulation.

**Figure S1.** (A) Reflectivity of InAs metasurface in different bias ranging from -2.5 V (depletion) to 2.5V (accumulation). (B-D) Electric field distribution at the three representative conditions, i.e. depletion, no-bias and accumulation for the applied voltages of -2.5 V, 0 V and 2.5 V, respectively. .

We start by investigating the reflectivity of proposed tunable metasurface with respect to different applied voltage. Finite-difference time-domain (FDTD) simulation is employed to monitor the intensity of reflected wave. Fig S1 A shows the set of reflection spectra for the applied voltage ranging from -2.5V (depletion) to +2.5V (accumulation).

For the bias between -2.5 V (depletion) to 0 V (no-bias), there exists a rapid shift of reflection dip from 5.4$\mu m$(depletion) to 5$\mu m$ (no-bias). Increased of electrical bias (-2.5 V to 0 V) leads to a decrease of the epsilon-near-zero (ENZ) wavelength in InAs, and this change gives a blue-shift of the reflection dip. Images of field distributions for the depletion and accumulation (Fig. S1 B, C) illustrates effective field confinement by the excitation of gap plasmon under the Al grating.

On the other hand, for the bias between 0 V (no-bias) to 2.5 V (accumulation), the reflection dip switches the movement between red- and blue-shifts. This is because in the accumulation state, the plasma frequency changes significantly due to a small effective mass of ideal InAs, which leads to the gigantic changes of ENZ and negative-valued real part of the permittivity in the targeted wavelength. The electric field intensity distribution at the wavelength of 5.0 μm is depicted in Figure S1 D. The field is largely confined within the accumulated region of InAs near the interface of Al_2_O_3_ due to the effect of ENZ and negative-valued permittivity.

From the analysis of ideal electrical bias sweeping, it is confirmed that the designed metasurface for reflectivity modulation effectively operates at the bias range between -2.5 V (depletion) and 0 V (no-bias), with which we demonstrate the reflectivity modulation by more than 40% in main text Fig 3.

**Figure S2.** Analysis of reflection spectra for the three representative biases of -2.5 V (A-D), 0V (E-H) and 2.5 V (I-L) with parametric sweeping of period (A,E,I), width (B,F,J), spacing (C,G,K) of metallic grating and thickness of oxide (D,H,I).

In order to optimize the electrically tunable metasurface, we analyze the reflection spectra for the different bias cases using Finite-difference time-domain (FDTD) simulation with various parametric sweeping of period, width, spacing of metallic grating and thickness of oxide. Figure S2 shows the reflectivity spectra for the depletion (-2.5 V) (Fig. S2 A-D), no-bias (0 V) (Fig. S2 E-H) and accumulation (2.5 V) (Fig. S2 I-L). As the period and width increases, the reflection dip shifts to longer wavelength due to the spectral change of resonant condition. (Fig. S2 A,B). In addition, spectrally separated dips at the accumulation condition merge into a single dip as the ENZ point shifts to the longer wavelength at depletion condition. From the analysis, it is found that the metasurface for reflectivity modulation operates efficiently at the optimally designed structural dimensions (*w: 660 nm*, *s: 50 nm*, *d*_Al2O3_: 5 nm, *d*_Al_: 50 nm, *d*_InAs_: 15 nm) and the targeted wavelength (*5~5.5*$\mu m$*)*.

**Supplementary note 2.** Applications of dynamic focusing and beam steering.

**Figure S3.** Reflectivity distributions of meta-atoms along the off-axis of grating direction (x-direction) to implement the dynamic Fresnel Zone plate (A-C). Intensities of electric field distribution for dynamic focusing at three representative focal length.

To realize the dynamic focusing in metasurface based Fresnel Zone plate, we conduct full field simulations by the meta-atom wise control of electrical bias. Fig. S3 A-C shows the reflectivity distributions at the Fresnel Zone plate for various focal length under the illumination with transverse magnetic (TM) polarization. The designed focal lengths are 100, 200 and 400$\mu m$, respectively. Fig. S3 D-F shows the simulated intensity (|E|^2^) distributions of the designed Fresnel Zone plate at x-z plane, which are consistent with the theoretical prediction.

**Figure S4.** Phase modulation in the InAs metasurface for dynamic wavefront shaping. The optimized metasurface shows relatively high-phase modulation (over 180°) at the wavelength of 5.16 $\mu m$.

**Figure S5.** Three representative designs of phase distribution in each super cell for various beam-steering angles.

To realize efficient beam steering, we control the phase of InAs metasurface to simulate various steering angles under the illumination with TM polarization. Fig. S5 A-C shows phase distribution in each super cell with period of phase composed of 6, 18, 24 antennas, respectively. 2D far-field patterns given in polar coordinates for different steering angles are presented in Fig. S5 D-F. When the metasurface consists of 6 super cells in each phase period, it leads to a reflection with main lobe up to 70°.
